# A Giant Right Heart Thrombus-in-Transit: The Challenge of Anticoagulation in Factor V Leiden Thrombophilia

**DOI:** 10.1155/2018/9098604

**Published:** 2018-09-27

**Authors:** Andrew Chu, Thu Thu Aung, Minni Shreya Arumugam, Mauricio Danckers, Mohi Mitiek, Jonathan Leslie

**Affiliations:** ^1^Internal Medicine Residency Department, Aventura Hospital and Medical Center, 20900 Biscayne Boulevard, Aventura, FL 33190, USA; ^2^Internal Medicine Residency Department, SUNY Upstate Medical University, 750 East Adams Street, Syracuse, NY 13210, USA

## Abstract

Factor V Leiden (FVL) is an autosomal dominant condition resulting in thrombophilia. Factor V normally acts as a cofactor for prothrombinase, helping cleave prothrombin to thrombin. A single point mutation in it disrupts factor V, making it unreceptive to protein C and increasing the risk of thrombosis. FVL mutation associated with right heart thrombus is a rare entity. Right heart thrombus or right heart thrombus-in-transit is associated with high mortality. We present a 51-year-old male with a past medical history of FVL homozygous mutation and recurrent blood clots, who has failed multiple different oral anticoagulants. He presented to the hospital with symptoms of shortness of breath and subsequently found to have a giant right heart thrombus. He was treated with surgical embolectomy. This case underscores the challenges faced by patients with FVL and recurrent blood clots.

## 1. Introduction

Right heart thrombus-in-transit (RHTT) is a rare occurrence and is associated with high mortality if left untreated. Increasing use of echocardiography has helped identify this disease early. RHTT associated with FVL thrombophilia proved to be a great challenge for clinicians due to the lack of strong medical evidence. No long-term anticoagulation is recommended in asymptomatic FVL homozygotes or heterozygotes [1]. For FVL patients, who have an active clot, long-term anticoagulation is recommended. The duration of treatment is decided on individual basis. We report a case of giant RHTT in a 51-year-old man with FVL mutation and recurrent blood clots who had been on multiple different oral anticoagulants. This case hopes to bring out the topic of RHTT and raises the challenges of anticoagulation in such individuals.

## 2. Case Presentation

A 51-year-old male was admitted to our hospital with a three-day history of shortness of breath. He mentioned of dry cough associated with chest discomfort. The pain was localized to the mid-sternum, nonradiating, exacerbated in supine position, and improved with sitting. He was taking aspirin at home for his symptoms. He denied history of sick contact, fever, chills, weight loss, night sweat, diaphoresis, palpitation, or dizziness.

He had an extensive past history including FVL homozygous mutation, recurrent lower extremity deep venous thrombosis (DVT) with inferior vena cava (IVC) filter placed, congestive heart failure requiring automatic implantable cardioverter-defibrillator (AICD) placement, and hypertension.

The patient was initially diagnosed with FVL mutation when he had his first episode of lower extremity DVT in 2002. At that time, he was placed on warfarin therapy with a goal international normalized ratio (INR) of 2-3. In 2007, he had a recurrent episode of lower extremity DVT and bilateral pulmonary embolism (PE) despite being compliant with warfarin and close INR monitoring. His INR on admission was 2.1. During admission, he had an IVC filter placed and his goal INR was increased to 2.5–3.5. In 2015, he had another recurrent lower extremity DVT despite having a higher target INR of 3.2. Warfarin was switched to rivaroxaban 15 mg twice a day for 21 days followed by 20 mg once daily. He denied a family history of malignancy or thrombophilia. He did undergo computed tomography (CT) of the abdomen and pelvis in 2015 which did not show gross evidence of intra-abdominal or pelvic mass. CT of the chest did not show evidence of pulmonary nodule.

**Figure 1:**
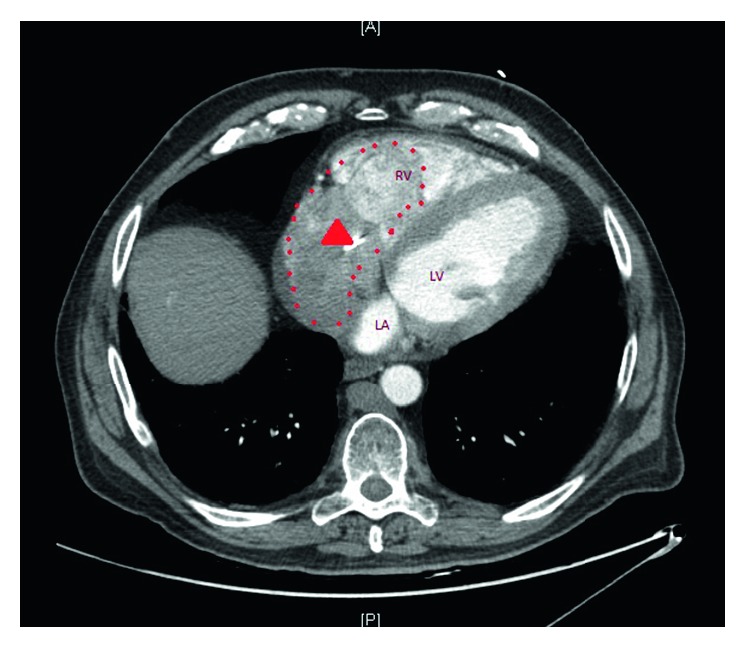
CT angiography of thorax displaying a suspected mass (dotted line) in the right side of the heart embedding the defibrillator wire (in red arrow head).

**Figure 2:**
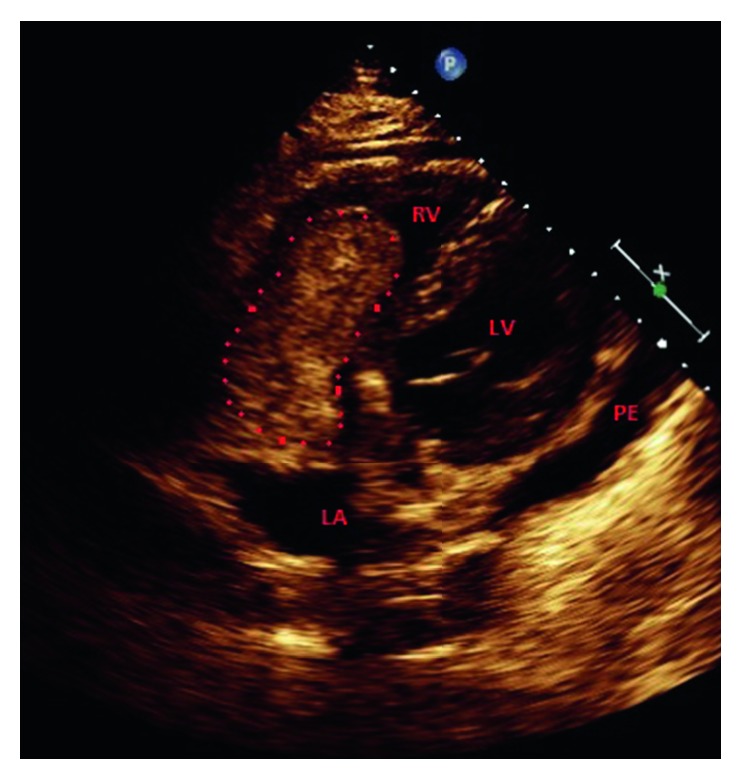
Transthoracic echocardiogram apical four chamber view showing the mobile mass (dotted line) within the right side of the heart. RV, right ventricle; LV, left ventricle; LA, left atrium; PE, pericardial effusion.

**Figure 3:**
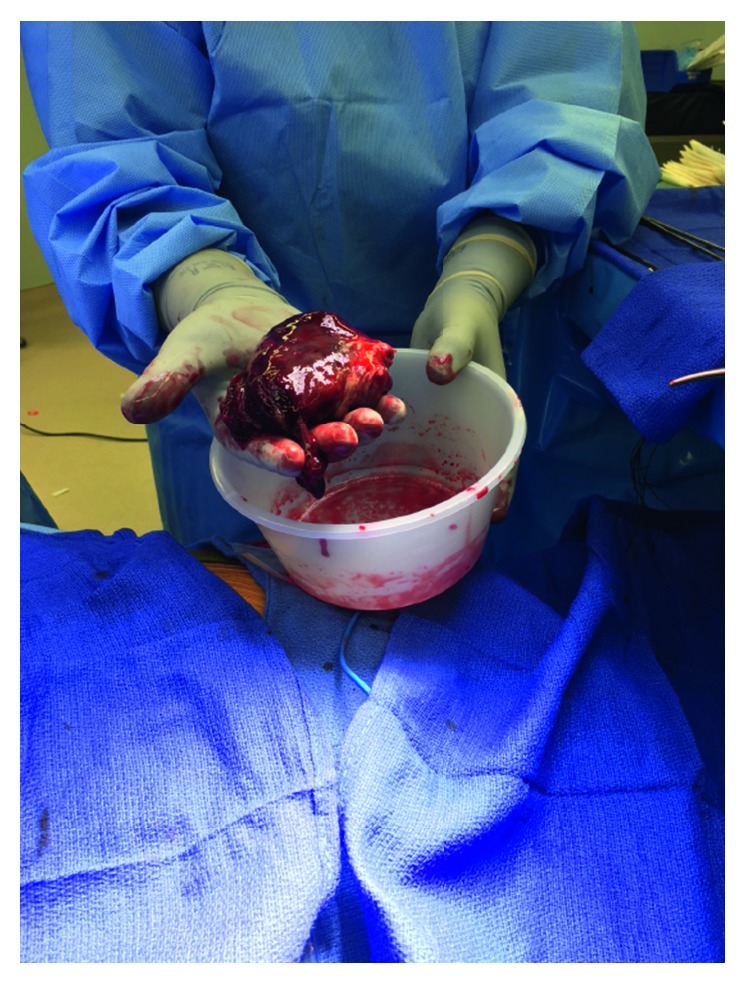
The giant right heart thrombus from surgical embolectomy.

The patient was taking aspirin 81 mg daily, atorvastatin 40 mg daily at bedtime, carvedilol 3.125 mg twice daily, lisinopril 5 mg daily, furosemide 40 mg daily, and rivaroxaban 20 mg once daily. The vital signs on admission were 99.5°F, heart rate 130 beats per minute, blood pressure 120/75 mmHg, respiratory rate 18 breaths per minute, and oxygen saturation 94% on 32% fraction of inspired oxygen. Physical examination was remarkable for distend jugular vein and crackles at bilateral lung bases. The results of blood work including complete blood count and comprehensive metabolic panel were within normal limits. Blood urea nitrogen was 19 mg/dL and serum creatinine was 0.9 mg/dL. Work up for autoimmune diseases came back negative. His prothrombin time was 26.1 seconds, activated partial thromboplastin time was 31.9 seconds, and the international normalized ratio was 2.35. Anterior-posterior chest X-ray illustrated a normal pattern. Venous Doppler of lower extremities revealed acute nonocclusive extensive thrombosis involving the bilateral common femoral, femoral, and popliteal veins. CT of the chest with contrast showed absence of pulmonary embolism, questionable right ventricular mass, moderate-sized pericardial effusion, and reflux of iodine contrast into the IVC and hepatic veins, suggesting right heart strain (Figure 1). Transthoracic echocardiogram showed ejection fraction between 25 and 30%, moderate diffuse hypokinesis of the left ventricle, and dilated left atrium. There was a definite, large, echogenic, highly mobile mass measuring 5.29 cm × 8.61 cm, up to 9.1 cm in length. The mass extended from the right atrium to the right ventricle, moving back-and-forth across the tricuspid valve (Figure 2). Ultrasound of IVC showed patent IVC filter without clots. He was taken for emergent right atrial and ventricular embolectomy. The thrombus was found to be originating from the coronary sinus (Figure 3). There were a small amount of clots around the AICD wires which were removed. Repeat transthoracic echocardiogram showed no mass, but mildly dilated right atrium and ventricle with trace pericardial effusion. The patient was then started on unfractionated heparin drip.

On postoperative day 11, the patient underwent sternal wound re-exploration due to gradual downward trend of his hemoglobin. He had a sternal dehiscence during which an extensive clot was found despite being on anticoagulation with unfractionated heparin drip. Heparin-induced antibody was negative. He had the clot removed surgically. The patient continued to improve and transitioned from unfractionated heparin drip with target PTT 50–60 seconds to dabigatran 150 mg oral twice a day. He was subsequently discharged to cardiac rehab facility and has been doing well.

## 3. Discussion

Right heart thrombus-in-transit (RHTT) occurs in 4% of pulmonary embolism. It can originate and be dislodged from extremities DVT, form in situ due to structural heart disease, or sometimes be associated with hardware such as pacemakers and prosthetic valves. The overall mortality rate in patient with RHTT is 28% [2]. Management of RHTT includes anticoagulation, systemic thrombolysis, catheter-directed intervention, or surgical embolectomy. In the recent meta-analysis published by Athappan et al. [3], surgical embolectomy has the greatest mortality benefit compared to systemic thrombolysis or anticoagulation alone (13.9%, 18.3%, and 37.1%, respectively). Very limited medical literatures have been published regarding management of RHTT in patients with FVL mutation.

FVL mutation is the most common inherited hypercoagulable state. It occurs due to a single point mutation on chromosome one, a G-to-A substitution leading to an amino acid replacement. This mutation leads to the inability of protein C to cleave and degrade factor V, leading to increased tendency to form blood clots [4]. Individuals who are heterozygous for FVL mutation have three to eightfold increased risk of venous thrombosis. The risk increased to 18- to 80-fold in individuals who are homozygous for FVL mutation [5]. Hence, the probability of developing an intracardiac thrombus would be higher in patients with hypercoagulable disorders than the general population. In addition to inherited hypercoagulability, intracardiac foreign bodies such as pacemakers and prosthetic valves have been associated with right heart thrombus.

No treatment is warranted for asymptomatic patients with FVL. For individuals with first venous thromboembolic event, anticoagulation therapy is generally administered for 6 months [4]. The treatment for venous thromboembolism includes initial anticoagulation with intravenous unfractionated heparin or subcutaneous low-molecular-weight heparin followed by oral anticoagulation with warfarin (target INR between 2 and 3). Prolonged duration with anticoagulation may be considered for certain individuals with increased recurrence risk of venous thromboembolism and uncertain nature of the index of venous thromboembolism [6]. However, any decision regarding the ideal duration of therapy must take into account the risk of bleeding with prolonged anticoagulation. FVL heterozygosity or prothrombin G20210A should not influence decisions about duration of anticoagulation therapy [4]. For individuals who developed recurrent venous thromboembolism while on warfarin, and their INR was within the target range of 2 and 3, an increased target INR or switching to a different oral anticoagulant can be considered. This must be done after weighing the risks and benefits. However, there is no medical literature to support this practice as events of recurrent venous thromboembolism are rare in FVL. Most importantly, clinicians must ensure patients are compliant with oral anticoagulation therapy.

Newer oral anticoagulants such as direct thrombin inhibitor (dabigatran) and factor Xa inhibitors (rivaroxaban, apixaban, and edoxaban) can be considered for patients who developed recurrent venous thromboembolism despite being compliant with warfarin therapy. These medications do not require frequent blood monitoring or dose adjustment. They also have lower interaction with other medications. Currently, there are reversal agents for dabigatran and rivaroxaban, should there be a need of one. There are currently no available data regarding the role of these newer oral anticoagulants in FVL.

Our patient with FVL homozygosity had a history of multiple recurrent venous thromboembolic events despite being on warfarin therapy and good medication compliance. He presented once again with another recurrent venous thromboembolism despite being on rivaroxaban. Therefore, we decided to anticoagulate our patient with dabigatran which has different pharmacologic mechanisms from warfarin and rivaroxaban.

Several cases have been reported regarding inheritable hypercoagulable states and RHTT. Nagae et al. reported a right ventricular mass in a 14-year-old girl who subsequently diagnosed with familial heparin cofactor II deficiency [7]. Corre et al. reported a case of coronary sinus thrombosis due to FVL [8]. Hajsadeghi et al. reported a 36-year-old man presented with productive coughs and hemoptysis [9]. He was subsequently found to have a right ventricular thrombosis and diagnosed with FVL.

Our patient's right-sided intracardiac thrombus is the largest ever reported associated with FVL. Significant attention was brought to our treatment team on how much hemodynamic compensation our patient has developed despite the thrombus causing almost-total-occlusion of blood flow from the right atrium to the right ventricle. Hemodynamic compensation was largely due to hypermobility of the RHTT, preventing the total occlusion of blood flow through the tricuspid valve. Our patient also had acquired factors for thrombophilia including low blood flow state due to underlying cardiomyopathy and presence of AICD wires and IVC filter which were the nidus for thrombosis. We believe that the patient's underlying inherited thrombophilia played the greatest role in developing this massive RHTT. The choice of anticoagulation continues to be the challenge in our patient as he had failed on two different anticoagulants in the past.

## 4. Conclusion

We have known inherited thrombophilia for several decades. However, the role of anticoagulation and different anticoagulants continues to be mysterious as there are no clear guidelines. This becomes a more challenging subject when dealing with RHTT, a near fatal disease if not managed properly. More studies are required to define the roles of different anticoagulants in inherited thrombophilia.
